# Nobiletin Delays Aging and Enhances Stress Resistance of *Caenorhabditis elegans*

**DOI:** 10.3390/ijms21010341

**Published:** 2020-01-04

**Authors:** Xueyan Yang, Hong Wang, Tong Li, Ling Chen, Bisheng Zheng, Rui Hai Liu

**Affiliations:** 1 Overseas Expertise Introduction Center for Discipline Innovation of Food Nutrition and Human Health (111 Center), School of Food Science and Engineering, South China University of Technology, Guangzhou 510641, China; yangxuexue1996@163.com (X.Y.); febzheng@scut.edu.cn (B.Z.); 2 Ministry of Education Engineering Research Centre of Starch & Protein Processing, Guangdong Province Key Laboratory for Green Processing of Natural Products and Product Safety, South China University of Technology, Guangzhou 510641, China; felchen@scut.edu.cn; 3 Department of Food Science, Stocking Hall, Cornell University, Ithaca, NY 14853, USA; tl24@cornell.edu; 4 Guangdong ERA Food & Life Health Research Institute, Guangzhou 510670, China

**Keywords:** nobiletin, aging, stress resistance, *Caenorhabditis elegans*

## Abstract

Nobiletin (NOB), one of polymethoxyflavone existing in citrus fruits, has been reported to exhibit a multitude of biological properties, including anti-inflammation, anti-oxidation, anti-atherosclerosis, neuroprotection, and anti-tumor activity. However, little is known about the anti-aging effect of NOB. The objective of this study was to determine the effects of NOB on lifespan, stress resistance, and its associated gene expression. Using *Caenorhabditis elegans*, an in vivo nematode model, we found that NOB remarkably extended the lifespan; slowed aging-related functional declines; and increased the resistance against various stressors, including heat shock and ultraviolet radiation. Also, NOB reduced the effects of paraquat stressor on nematodes and scavenged reactive oxygen species (ROS). Furthermore, gene expression revealed that NOB upregulated the expression of *sod-3*, *hsp-16.2*, *gst-4*, *skn-1*, *sek-1*, and *sir-2.1*, which was suggested that anti-aging activity of NOB was mediated most likely by activation of the target genes of the transcription factors including dauer formation (DAF)-16, heat-shock transcription factor (HSF)-1, and skinhead (SKN)-1. In summary, NOB has potential application in extension of lifespan, and its associated healthspan and stress resistances.

## 1. Introduction

The global population is stepping into the stage of aging. By 2050, 16% of people in the world will be over the age of 65, up from 9% in 2019, and the number of persons aged 80 years or over is projected to triple, from 143 million in 2019 to 426 million in 2050 [1]. With an increasing proportion of the elderly in society and, consequently, rising incidences of aging-associated diseases, it has become more and more urgent to seek nutraceuticals, not only to prolong lifespan, but also to improve healthspan as well as stress resistance. Thousands of phytochemicals, present in plant-derived food, such as vitamin C, phenolics, and flavonoids, play an important physiological role in the maintenance of human health. Epidemiological studies have shown that increasing the intake of fruits and vegetables rich in phytochemicals can protect the body from chronic diseases such as cancer, atherosclerosis, and neurodegenerative diseases in an age-dependent manner [2,3,4]. In addition, similar improvement on cognitive performance in the elderly could be achieved through consumption of flavonoid-rich food like wine, tea, and chocolate [5].

The short-lived model organism *Caenorhabditis elegans* (*C. elegans*) offers promising possibilities for studying the influence of bioactive compounds on the process of aging, given that its longevity-associated signaling pathways are highly conservative to higher mammals, including humans [6]. Additionally, some aging-related physiological indexes occurring in this kind of worm, are similar to those found in higher mammals. In *C. elegans*, the insulin/- insulin-like growth factor signaling (IIS), heat-shock transcription factor (HSF-1), mitogen-activated protein kinase (MAPK), and dietary restriction (DR) pathways have been proved to be involved in aging and the onset of aging-associated diseases [7,8]. The IIS pathway, an evolutionarily conserved central control pathway similar to the human aging process, plays an important role in the regulation of lifespan in both nematodes and mammals by regulating the conserved transcription factor DAF-16 [9,10,11]. The HSF-1 pathway prolongs longevity by activating heat shock gene expression [12], while MAPK upregulates SKN-1, the nuclear factor-E2-related factor 2 (Nrf-2) homologue transcription factor, to combat stress-induced aging [13]. These three transcription factors are critical for stress resistance and longevity via promoting expression of antioxidant or detoxifying enzymes. Previous studies have reported that various flavonoids and extracts rich in flavonoids exert life-prolonging effects through the inhibition of the IIS pathway. For instance, quercetin [14,15] was found to prolong the lifespan of *C. elegans* by 10–15%, and promote stress resistance through rapid translocation of DAF-16 and activation of the downstream targets of DAF-16. Similarly, the anti-aging effect of myricetin was partly dependent on DAF-16 through a nuclear translocation of DAF-16, but not of SKN-1 [16]. Previously, our lab [17] reported that blueberry extract rich in polyphenols extended lifespan and increased survival rates after acute stress through the regulation of downstream gene of *daf-16* in *C. elegans*. Besides, the oxidative stress resistance promoted by pasteurized orange juice (POJ) treatment in *C. elegans* has been reported as a result of the transcription factor SKN-1 action [18].

**Figure 1:**
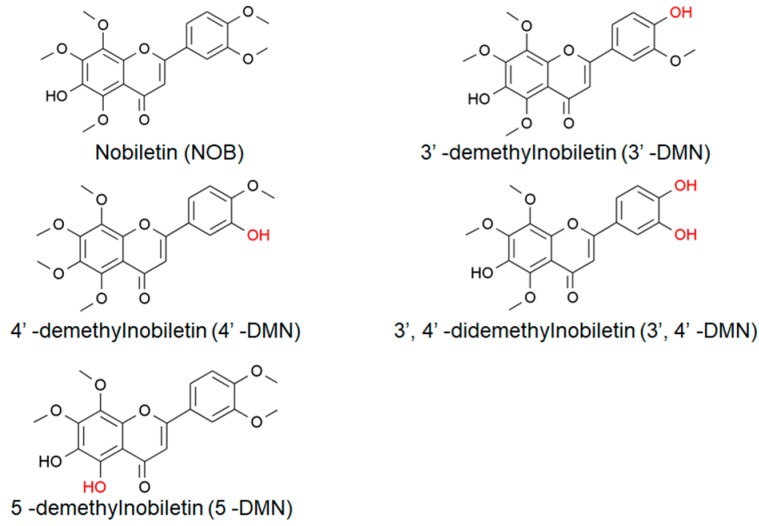
Chemical structures of nobiletin (NOB) and its derivatives.

Nobiletin (5,6,7,8,3′,4′-hexamethoxyflavone, NOB, Figure 1), one of polymethoxylated flavones (PMFs) extracted exclusively in citrus fruits, is abundant in *Citrus reticulate* (mandarin oranges), *Citrus tangerine* (tangerines), *Citrus sinensis* (oranges), and *Citrus aurantium* (grapefruits) (Table 1) [19,20,21], and as reported, *Citrus tangerine* contains the highest the content of NOB. After undergoing metabolism or autohydrolysis, many different derivatives will be generated, the most common of which are the following four: 3′-demethylnobiletin (3′-DMN), 4′-DMN, 3′,4′-DMN, and 5–DMN (Figure 1) [22,23,24]. Recently, NOB has become a hotspot as one of candidates for the prevention of Alzheimer’s disease (AD), which is a chronic and devastating progressive age-associated neurodegenerative disease. Nakajima et al. [25] have shown that NOB improved the pathological features of AD. Qi et al. [26] reported on the protective effects of NOB on neuroinflammation and cognitive deficits of three-month old mice. Recently, Nohara et al. [27] discovered that mice fed with normal diets with NOB lived longer than the control group by more than one month at 50% death. These results attract our attention, and whether NOB has the effect of extending lifespan and enhancing healthspan is worth further study.

In this work, we assessed the effect of NOB on longevity and its protective effect against multiple stressors using *C. elegans* and examined the potential molecular mechanisms underlying lifespan extension by NOB at the transcriptional level.

## 2. Results

### 2.1. Nobiletin (NOB) Increased Lifespan of C. elegans

**Figure 2:**
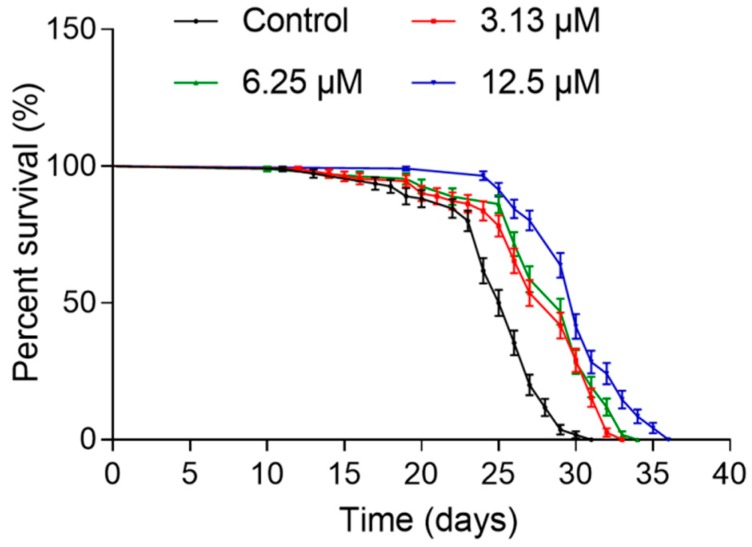
Effect of nobiletin (NOB) on lifespan of *C. elegans.* N2 worms were treated without NOB (control) or with NOB (3.13, 6.25 or 12.5 µM) from day 0 adult (L4 larvae) to death at 20 °C. The percentage of live animals was plotted against adult days. Detailed parameters are presented in Table 2.

To evaluate the prolongevity effects of NOB, we examined the lifespan of *C. elegans* treated with different concentrations of NOB, as shown in Figure 2 and Table 2. The mean lifespan of *C. elegans* treated with NOB at 3.13, 6.25, and 12.5 μM significantly increased to 27.06 ± 2.06 days (maximum lifespan: 33 days), 28.61 ± 0.07 days (maximum lifespan: 34 days), and 30.14 ± 0.28 days (maximum lifespan: 36 days), respectively, compared with that of DMSO-treated worms (mean lifespan: 24.9 ± 0.4 days, maximum lifespan: 31 days) (Table 2). There were 8.70%, 14.9%, and 21.0% increases in the mean lifespan of worms treated with 3.13, 6.25, and 12.5 μM of NOB, respectively, in comparison with the control group. Taken together, NOB effectively extended the lifespan of *C. elegans* in a dose-dependent manner.

### 2.2. NOB Delayed Aging-Related Functional Decline of C. elegans

**Figure 3:**
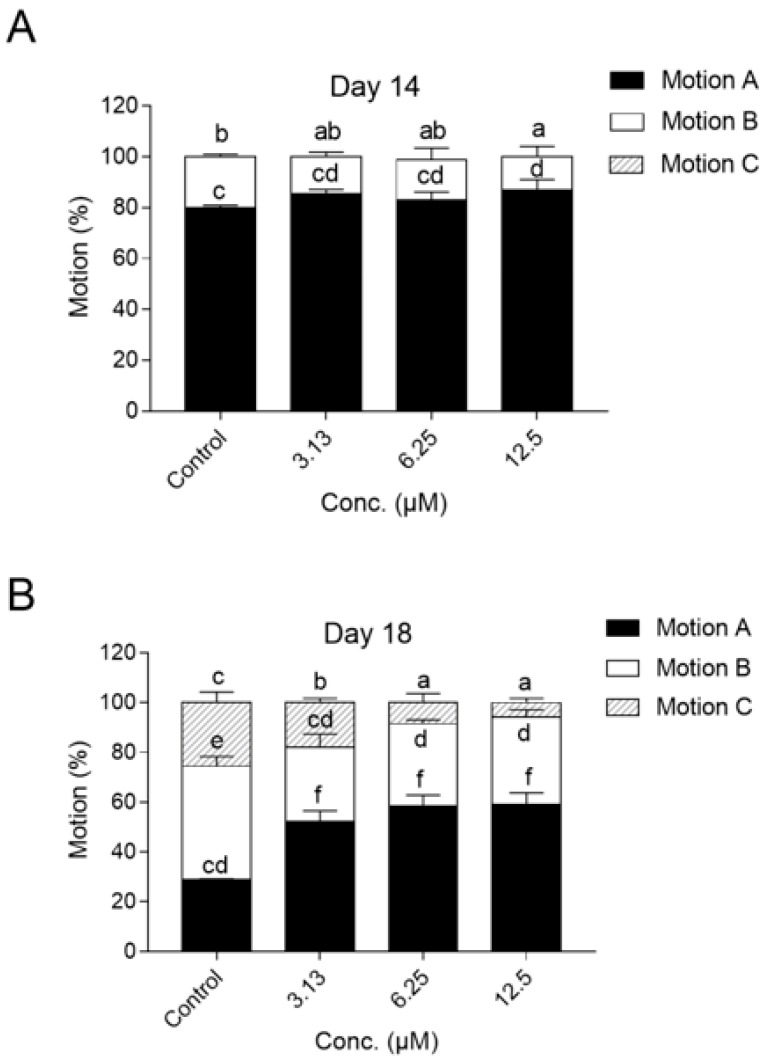

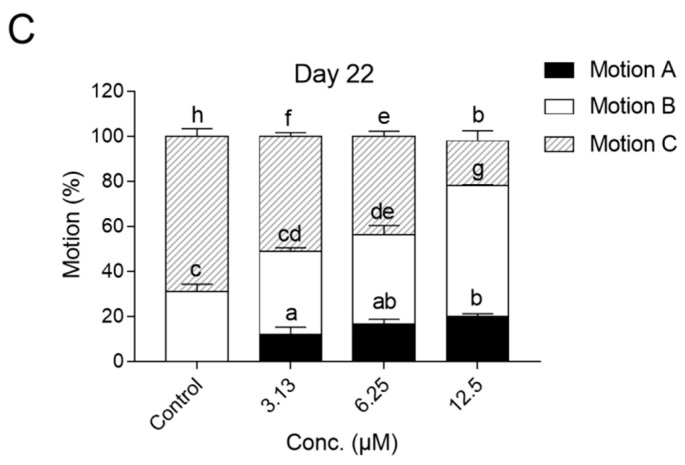
Effect of NOB on motility of (**A**) day 14, (**B**) day 18, and (**C**) day 22 experimental animals. Nematodes were classified into three groups based on motility: group A animals moved spontaneously and freely; group C animals only moved their heads in response to stimulating heads and tails; and group B animals were distributed between these two situations. NOB potently alleviated the decline of in motility in a dose-dependent manner. (*n* ≥ 40 animals per group for days 14 and 18; *n* ≥ 25 for day 22). Bars with different letters indicated statistical significance (*p* < 0.05).

To further investigate whether the increase in lifespan was accompanied by the improvement of aging-related functional decline, we assessed the motility of day 14, 18, and 22 experimental animals. On day 14, the motility of most animals was not affected, and they could still swim spontaneously and freely, except that there was a significant difference between the control and high (12.5 μM) dose group (Figure 3A) (*p* < 0.05). On day 18, compared with 28.8% of high-motility (class A) worms in the control group, remarkably more worms (52.4%, 58.3%, and 59.2%, receptively) of high motility were observed in the groups treated with 3.13, 6.25, and 12.5 μM NOB (Figure 3B). By day 22, the motility of the worms in the NOB-treated groups could maintain better motility. Over 78.0% of worms in high-dose group (12.5 μM) remained A or B motility, while only 31.0% worms had class B motility in the control group. Overall, the decline with aging in motility of worms was delayed significantly in a dose-dependent manner after treatment with NOB.

**Figure 4:**
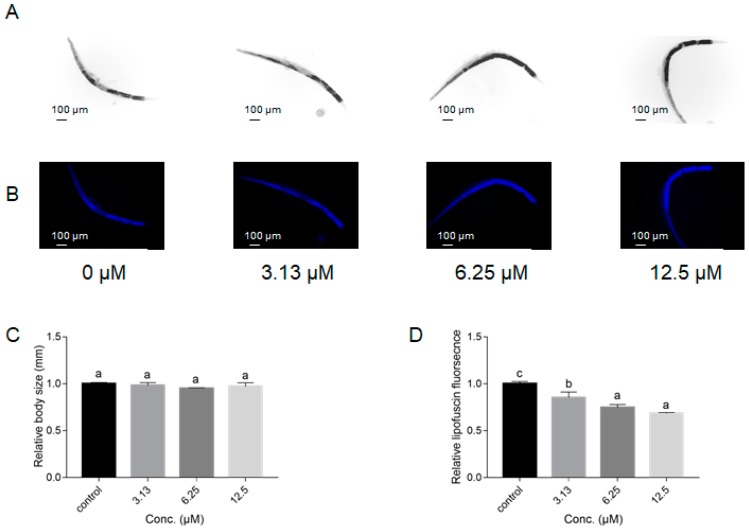
Effect of NOB on body size and lipofuscin accumulation in *C. elegans*. Wild-type N2 animals were treated without or with 3.13, 6.25, and 12.5 µM NOB for five days at 20 °C. The body size and lipofuscin accumulation were measured using fluorescence microscope, and the results are presented at (**A**,**B**). Body length and lipofuscin were quantitated by Image J software (**C**,**D**). Bars with different letters indicate statistical significance (*p* < 0.05).

Thereafter, other aging-related physiological changes including body size and lipofuscin accumulation were assessed in *C. elegans*. In terms of body size (Figure 4A,C), there were no significant changes among NOB-treated groups and the control group. In contrast, lipofuscin accumulation in worms treated with 3.13, 6.25, and 12.5 μM of NOB was significantly decreased by 14.8%, 25.3%, and 31.4%, respectively, in comparison with the control group (Figure 4B,C). These results indicated that NOB has the potential to dose-dependently decrease lipofuscin accumulation.

### 2.3. NOB Had No Significant Effect on Fertility

**Figure 5:**
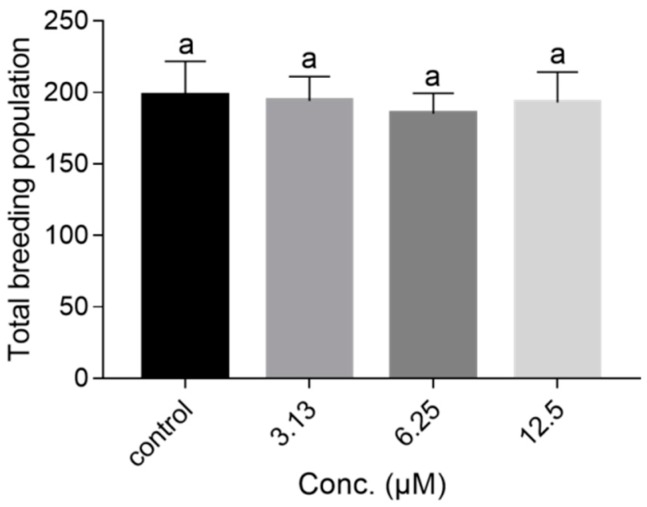
Effect of NOB on oviposition amount of *C. elegans* (*n* = 5 animals per group). There were no significant differences in the number of total progeny between NOB-treated and untreated animals (*p* < 0.05).

To examine the effect of NOB treatment on fertility, we counted the oviposition amounts of five worms in each group. As shown in Figure 5, the total number of offspring in all groups was all around 200, and there were no significant differences between NOB-treated groups and the control. Therefore, NOB had no significant influence on the reproductive capacity of *C. elegans*.

### 2.4. NOB Increased Resistance to Heat Shock, UV Radiation, and Oxidative Stress

Many studies have shown that an increase in lifespan is often correlated with enhancing resistance to stresses [28,29]. To assess the potential increased resistance of NOB, we performed several stress resistance assays in which pre-treated animals were exposed to various stressors, including heat shock at 35 °C, 1200 J/m^2^ of UV radiation, and 10 mM paraquat.

**Figure 6:**
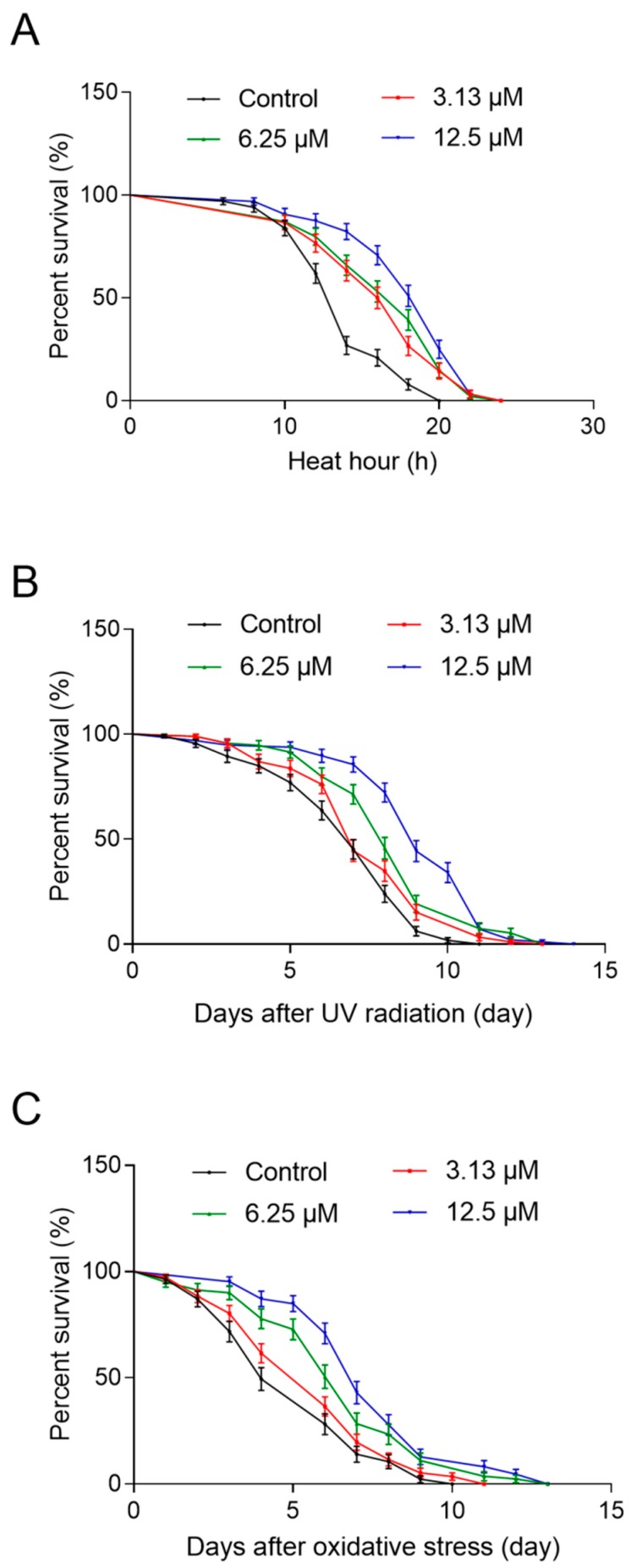
Effect of NOB on stress resistance of *C. elegans.* The adult nematodes were treated with 3.13, 6.25, and 12.5 µM NOB for five days at 20 °C, followed by three types of stressors. Compared with the control group, NOB significantly raised the resistance of nematodes to (**A**) heat shock at 35 °C, (**B**) UV damage, and (**C**) paraquat-induced oxidative stress. Detailed parameters are presented in Table 3, Table 4 and Table 5.

The results showed that thermotolerance was enhanced significantly by NOB treatment. We observed that animals treated with 3.13, 6.25, and 12.5 μM NOB showed a significantly increased maximum and mean lifespan, compared with untreated controls (Figure 6A, Table 3). The longest the mean lifespan was found in the high-dose group (18.10 ± 0.60 h), which was increased by 30.6% compared with the control group (13.86 ± 0.40 h).

Similar results were obtained in the UV radiation assay, animals treated with NOB showed improved UV radiation resistance compared with the control (Figure 6B, Table 4). Wild-type N2 animals treated with 6.25 and 12.5 μM NOB survived much longer than control animals, and the survival time was increased significantly by 20.5% and 33.3%, respectively.

We also performed the paraquat-induced oxidative stress resistance assay on N2 nematodes. Longer survival time was observed in the NOB-treated groups than in the control group. The mean survival time of worms treated with NOB at 3.13, 6.25, and 12.5 μM was 11.6%, 30.5%, and 47.8%, respectively, longer than that in the control group (Figure 6C, Table 5).

### 2.5. NOB Decreased ROS Accumulation In Vivo and Increased Antioxidant Enzyme Activities

**Figure 7:**
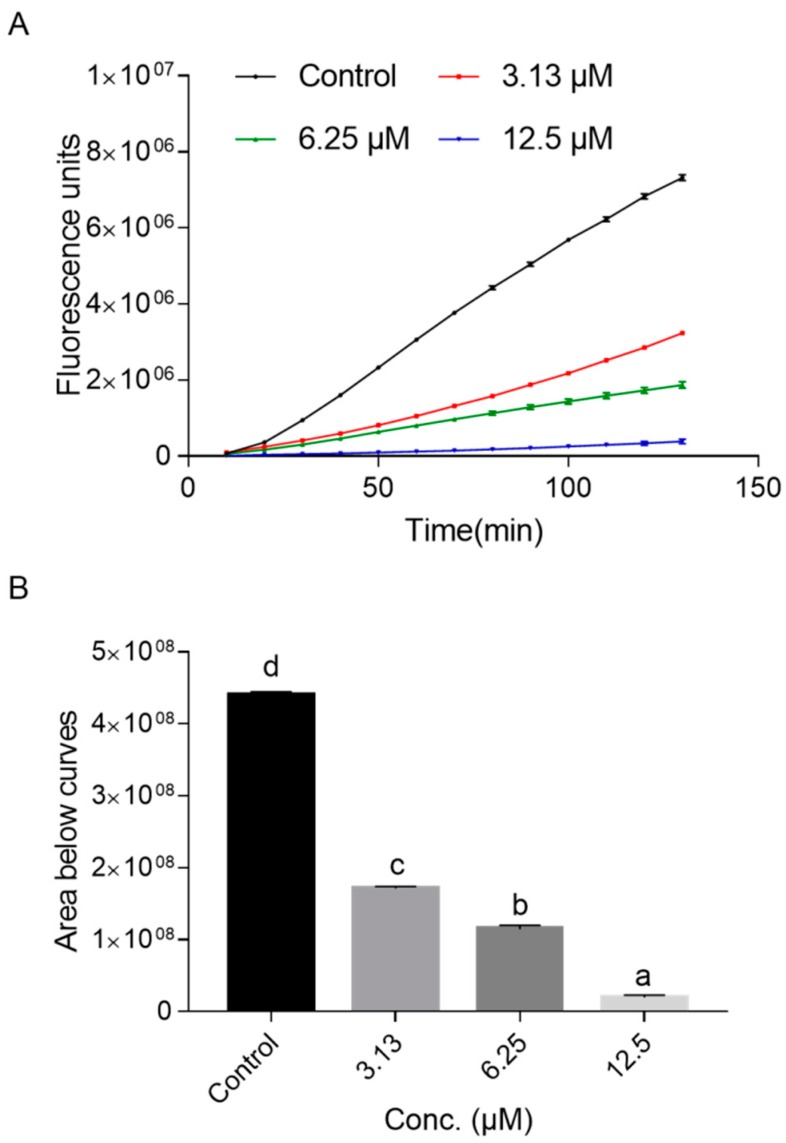
Inhibition of ROS production in *C. elegans.* The fluorescence curves against measuring time shown (**A**) were plotted from three independent trails, and the fluorescence protection area of NOB was shown according to the area under the curve (AUC) of the NOB treatment group and blank group (**B**).

**Figure 8:**
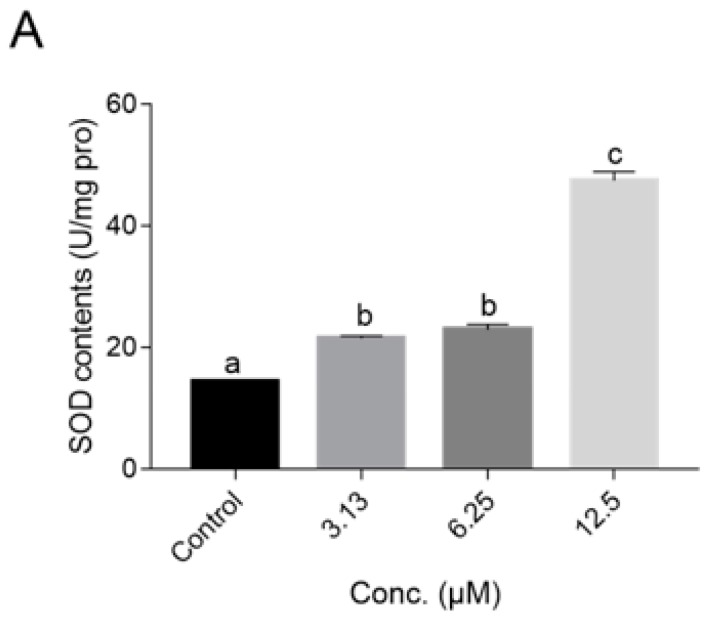

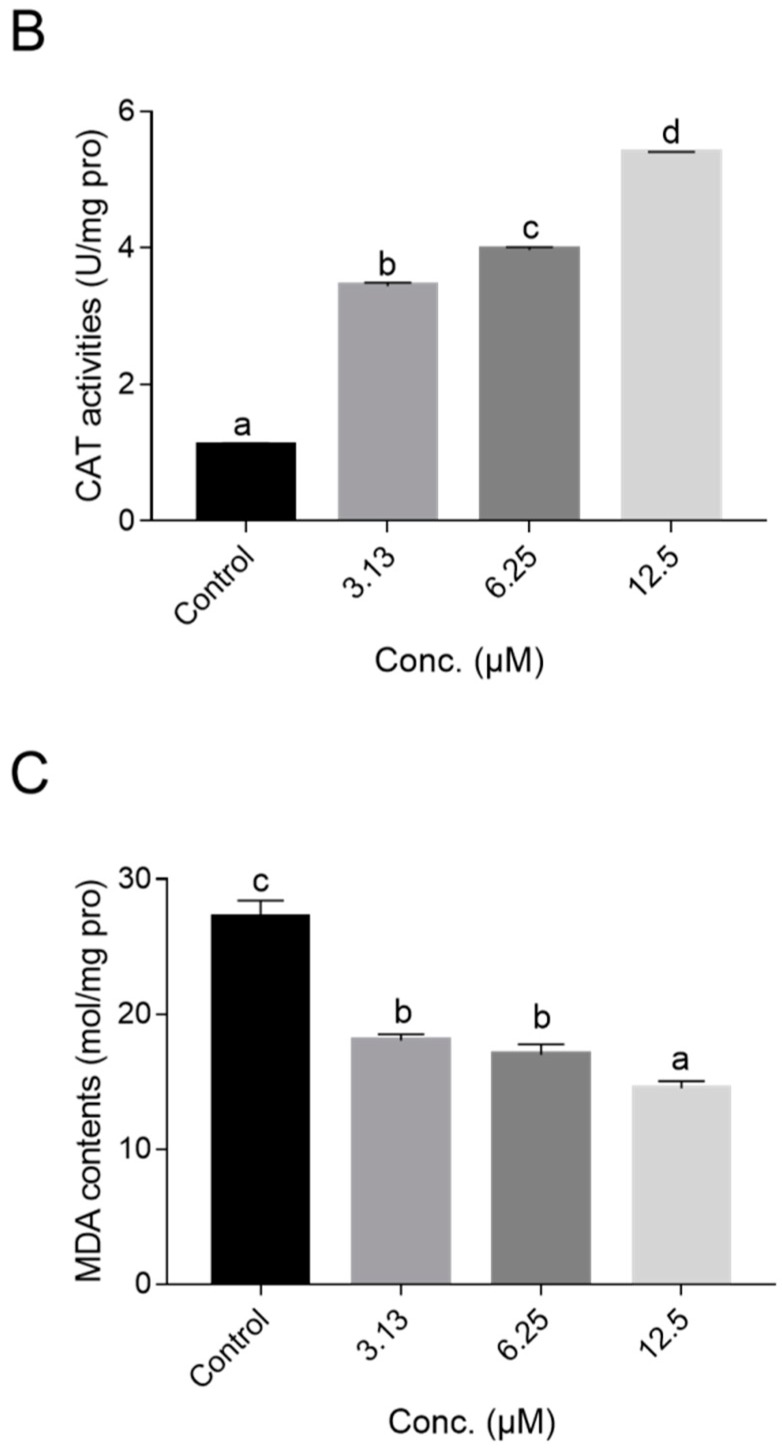
Effect of NOB on antioxidant enzymes in *C. elegans*. The activities of relative antioxidant enzymes: SOD (**A**) and CAT (**B**), and the malondialdehyde (MDA) content (**C**) in *C. elegans* after treatment with indicated concentrations of NOB. Data are shown as mean ± SD, *n* = 3. Bars with different letters indicated statistical significance (*p* < 0.05).

Next, we tested the influence of the NOB treatment on the intracellular accumulation of ROS and antioxidant enzyme activity. The results suggested that the ROS levels in *C. elegans* were significantly reduced in a dose-dependent manner after treated with NOB, as shown in Figure 7. Moreover, the activities of superoxide dismutase (SOD) and catalase (CAT) were also observed to increase markedly after NOB treatment (Figure 8). The high-concentration (12.5 μM) treatment group resulted in a more than tripling of activities, compared with the control. In addition, the malondialdehyde (MDA) contents after NOB treatment were reduced by more than 30% compared with that in the control group.

### 2.6. NOB Regulated the Expression Levels of Genes Involved in Life Extension and Stress Resistance

**Figure 9:**
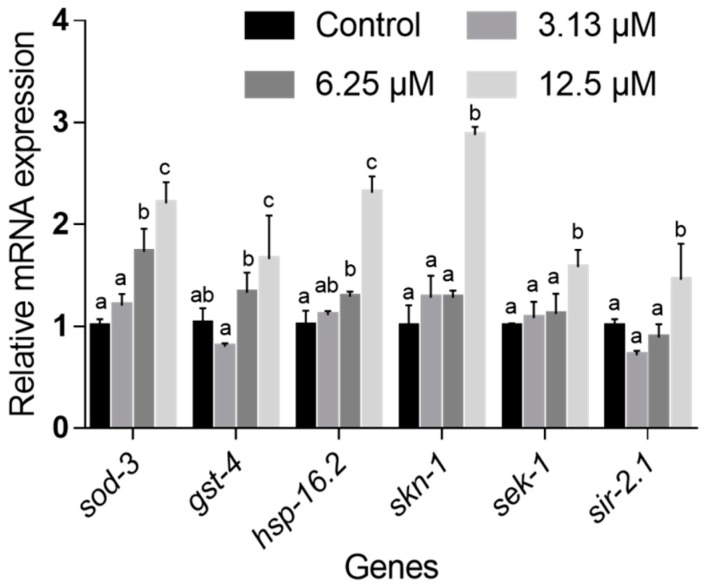
Effect of NOB on antioxidant gene expression in *C. elegans.* Data are shown as mean ± SD, *n* = 3. Bars with different letters in each group indicated statistical significance (*p* < 0.05).

To gain insight into the mechanism by which NOB extended the lifespan and enhanced stress resistance, we analyzed the mRNA expression of putative reporter genes related to stress resistance and longevity in N2 worms. As shown in Figure 9, compared with the control group, NOB treatment with 3.13, 6.25, and 12.5 μM up-regulated the relative expression levels of *hsp-16.2* to 1.11-, 1.29-, and 2.31-fold, respectively, and those of *sod-3* to 1.20-, 1.73-, and 2.21-fold, respectively. The expression of *gst-4* in the 3.13, 6.25, and 12.5 μM NOB treatment groups was 0.80, 1.33, and 1.66 times, respectively. Besides, we found that treatment with NOB at 12.5 μM activated the expression level of *skn-1*, *sek-1*, and *sir-2.1* (Figure 9), although there were no significant differences between the low and medium dose-treated groups. Generally, NOB treatment up-regulated the expression of *hsp-16.2*, *sod-3*, *gst-4*, *skn-1*, *sek-1*, and *sir-2.1*.

## 3. Discussion

Previous studies have shown that NOB has strong antimicrobial, antioxidant, anti-proliferative, anti-inflammatory, and anti-dementia activities [26,30,31,32,33,34,35]. Convincing evidence has shown that NOB promotes healthy aging in aged male mice with regular or high-fat diets, which works against metabolic disorders and aging-related energy imbalance, including restoring glucose homeostasis, promoting energy expenditure, cold tolerance and circadian activity, optimizing mitochondrial respiratory chain complexes (MRCs) activity and architecture in aged skeletal muscle, and regulating cholesterol and bile acid metabolism [27,36]. In this study, we further explored whether NOB has an effect on aging using the short-lived model organism, *C. elegans*. The results showed that NOB extended the mean and maximum lifespan of worms at certain concentration (3.13, 6.25, and 12.5 µM) in a dose-dependent manner (Table 2). Notably, the high-concentration treatment (12.5 µM) was found to enhance the mean lifespan of *C. elegans* (increased by 21.0%) to a higher extent compared with many kinds of flavonoids, and even achieve a comparable effect of some crude extracts of fruits and vegetables. For example, compared with the control group, quercetin (100 μM) increased the mean lifespan by 15% [14], and myricetin (100 μM) showed a similar life-prolonging effect with the value of 18% [37]. Additionally, Wang et al. [17] revealed that 50 mg/mL of blueberry extract mediated lifespan extension in *C. elegans* to 22.2%. After 2% ‘Cara Cara’ orange juice treatment, the mean lifespan of *C. elegans* was observed to extend by 29.5% versus the control group [18]. Considering toxicity or antimicrobial ability [38], it is possible that the NOB lengthened the lifespan via inhibiting bacterial growth. Delaney et al. came to the conclusion that NOB has no induction effect on cultured bacteria and no genotoxicity for mammals [39]. In order to exclude simple antimicrobial effects as longevity elicitor, we detected the inhibitory concentration of NOB by Oxford cup within a wide range of concentrations (10, 50, 100, 200, and 500 µM). However, no bacteriostasis was found at these concentrations (data are not shown). Thus, it is proved that the toxicity or antimicrobial hypothesis is unrelated to prolonging lifespan.

It was worth mentioning that NOB also exhibited obvious improved aging-associated physiological and structural parameters, such as motility and lipofuscin (Figure 3 and Figure 4). As shown in Figure 4D, the relative lipofuscin content in animals supplemented with 12.5 µM of NOB was only 75% of that in the control group. In contrast, treated animals maintained higher motility than those in the control group. Additionally, there were no significant differences of body size or reproductive output among the NOB-treated and control groups (Figure 4C and Figure 5), which demonstrates from another aspect that the concentration used does not have any toxicity. Kirkwood has reported that the extension of lifespan is accompanied by a decrease in other aspects (such as reproduction and growth rate) owing to a limited amount of energy of organisms, which is known as disposable soma theory [40]. Here, no significant differences of cumulative reproductive output or body size were caused by NOB treatment, which suggested that the effect of NOB might be independently in line with Kirkwood’s theory.

Many studies have shown that extension of the lifespan is accompanied with enhancing resistance to stress in *C. elegans* [17,41,42]. We also assessed whether NOB treatments improved *C. elegans* stress resistance to various stressors, including thermal stress, UV radiation, and oxidative stress. The results showed that NOB was able to exert stress protection to three environmental stressors (Figure 6, Table 3, Table 4 and Table 5), which was consistent with the observation that NOB alleviated oxidative damages in the senescence-accelerated mouse [30].

It was widely accepted that aging might be the result of damage to DNA, proteins, and lipids by excess ROS generated from normal metabolism or external stresses [43]. Besides, oxidative stress is an important risk factor for various diseases associated with aging, such as cardiovascular diseases, neurodegenerative disorders, and cancer [44,45,46]. Besides scavenging activity of free radicals, polyphenols also exert indirect antioxidant effects by elevating endogenous antioxidant expressions. For instance, quercetin and myricetin (each 100 μM) decreased ROS levels in *C. elegans* by 70% and 60% after 48 h of incubation, respectively [16,37]. Moreover, Zhou et al. [47] found that the resistance effect of didymin on UV radiation was related to lower ROS levels and higher SOD activity. The ability of NOB to quench ROS directly has been reported in cell models [48,49]. Here, the antioxidant capacity of NOB was further assessed in *C. elegans*, including changes in the level of the antioxidant metabolite status and the antioxidant enzyme activities. The results showed that NOB significantly attenuated intracellular ROS generation (Figure 7) and up-regulated the activities of antioxidant enzymes (SOD and CAT) and decreased the synthesis of oxidant metabolites (MDA) (Figure 8), which suggested that the antioxidant activities of NOB might contribute to its anti-aging effect.

**Figure 10:**
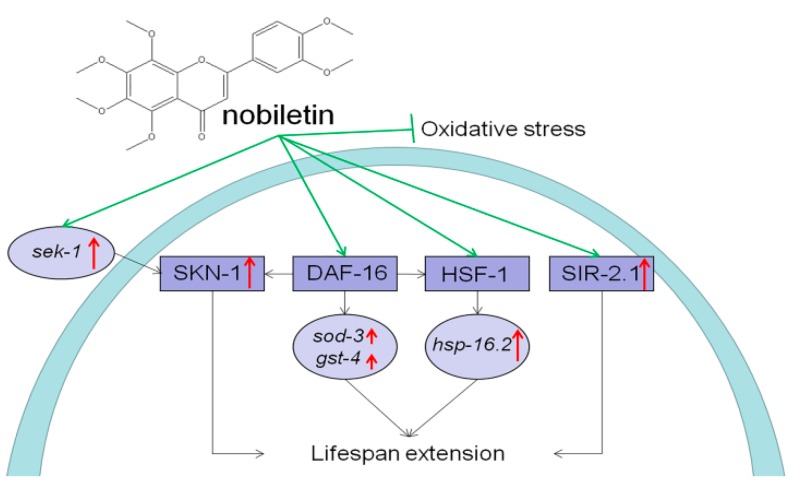
A possible mechanism of action through DAF-16 signalling pathway in *C. elegans* representing molecular targets for modulation by NOB.

Furthermore, several genes responsible for endogenous longevity and stress-response in wild-type nematodes according to a previous report [50] were selected to explore the effect of NOB treatment at the transcription level. The IIS pathway, which is an evolutionary conserved mechanism, plays a role in longevity and metabolism throughout different species. The initiation of the kinase cascade depends on the phosphorylation of DAF-2, the insulin/IGF-1 receptor in *C. elegans*, followed by the activation of several downstream genes, which shortens the lifespan of nematodes mainly by isolating the fork transcription factor DAF-16 in the cytoplasm (Figure 10). Contrariwise, the inhibition of the DAF-2 pathway causes nuclear localization and activation of DAF-16, and finally leads to enhancing the anti-aging ability of *C. elegans. Sod-3* (superoxide dismutase gene) and *gst-4* (catalase gene), located downstream of *daf-16*, play a protective role in stress resistance of *C. elegans* [9], which are commonly used to evaluate the modulation of the DAF-16 IIS pathway. As shown in Figure 9, the mRNA levels of *sod-3* and *gst-4* were both significantly increased in a dose-dependent manner after NOB-treatment (Figure 10), which indicated that the role of NOB in lifespan extension and improvement against stress of *C. elegans* might be attributed to activation of *sod-3* and *gst-4* genes through the DAF-16 IIS pathway. A previous study also found that the significant longevity-extending effects of epigallocatechin gallate (EGCG) could be mediated by the up-regulation of aging-associated genes such as *daf-16* and *sod-3* [51]. Similarly, the up-regulation mRNA level of *sod-3* by 1.77-fold was also detected upon 50 μM of apigenin treatment, which plays a protective role against stress resistance by promoting DAF-16 activity [52]. Heat shock proteins (HSP) could protect nematodes from stress by altering the activity of proteins required for resistance to thermal stress [29,53]. Members of the HSP-16 protein family in *C. elegans* are suggested as predictors for longevity, because they are closely linked to thermotolerance and longevity [29]. The expression of *hsp* genes is mainly regulated by HSF-1; besides, it is also influenced by IIS pathway. Thus, upregulation of *hsp-16.2* expression by NOB treatment might involve the HSF-1 pathway, leading to improvement of stress resistance and longevity extension.

In addition, the expression levels of *skn-1*, *sek-1*, and *sir-2.1* genes at a high concentration of NOB were increased by 2.88-, 1.58-, and 1.46-fold, although the variation was not significant at low and medium concentrations (Figure 9). The activation of the MAPK pathway leads to nuclear localization and activation of the transcription factor SKN-1, which regulates the expression of downstream genes, including antioxidant proteins such as SOD, glutathione-s-transferase (GST), or glycerol phosphate oxidase (GPO) and phase II detoxifying enzymes [54,55]. *Sek-1* plays an important role in resisting to stress by controlling DAF-16 and SKN-1 from cytoplasm to nucleus through the MAPK pathway [56,57]. Lin et al. [58] reported that 180 µM of carnosic acid (CA) increased the expression of *sek-1* 13.28-fold, which indicated that the lifespan extension mediated by CA might be dependent on the MAPK pathways. *Sir-2.1*, which encodes a histone deacetylase-like nicotinamide adenine dinucleotide (NAD)^+^-dependent protein that integrates metabolic status with lifespan, has been shown to be associated with the life-prolonging effects of some phenolics, such as resveratrol [59].

Herein, these results suggested that the role of NOB in prolonging life might be associated with three transcription factors, including DAF-16, HSF-1, and SKN-1 (Figure 10). Previous studies have indicated that there is a regulatory interaction among transcription factors. The expression of *hsp-16.2* gene is mainly regulated by HSF-1, but also influenced by the IIS pathway in *C. elegans* [12]. Tullet et al. [13] reported that the transcription network regulated by SKN-1 was also an important direct target of IIS, and DAF-16 could reduce the nuclear localization of SKN-1 to regulate its activity. Therefore, we suggest the hypothesis that NOB might go through the IIS pathway to extend lifespan and improve the stress resistance.

NOB is mainly derived from citrus plants, especially in the peels of *Citrus reticulate* and *Citrus sinensis*. Citrus peel, as the major byproduct produced, has been processed into a traditional herbal medicine in East Asia for several hundred years. Zhang et al. measured the efficiency of four dietary extraction methods of NOB from dried tangerine peel (DTP) [60]. The results have shown that the highest concentration of NOB was obtained by ethanol extraction, followed by soaking, boiling, and steaming. It provides a basis for the most appropriate utilization of citrus peels and for NOB consumption.

With a high degree of methylation, PMFs have better bioavailability owing to better lipid solubility than other polyhydroxyflavones and flavonoid glycosides [61]. Yuen et al. reported that NOB (13.30 mg/L) has good absorption in the whole intestine throughout rat unilateral intestinal perfusion model [62]. In addition, NOB promotes the body’s lipid metabolism, which in turn also promotes its own intestinal absorption, and reaches brain tissue at a high concentration [22,23,24]. Most pharmacokinetic reports on NOB have proven that the effects of NOB are achieved through demethylated derivatives produced after metabolism [63,64]. Further research on anti-aging effects can be carried out using these biologically active derivatives of NOB. Besides, research on in vivo metabolic kinetics will be conducted to provide information for the formulation and dosage design of NOB as nutraceuticals and functional food supplements.

## 4. Materials and Methods

### 4.1. Chemicals and Reagents

Nobiletin (NOB, ≧98%, HPLC-grade), dimethyl sulfoxide (DMSO), 5-fluoro-2′-deoxyuridine (FUDR, ≥99.9%,), 2′, 7′-dichlorofluorescein diacetate (DCFH-DA), and methyl viologen dichloride hydrate (paraquat) were purchased commercially from Sigma-Aldrich (St. Louis, MO, USA).

### 4.2. Nematode Strains and Culture Conditions

The *C. elegans* strain (Bristol N2, wild-type), obtained from Caenorhabditis Genetics Center (CGC, University of Minnesota, Minneapolis, MN, USA) was maintained at 20 °C on nematode growth agar medium (NGM) plates seeded with live *Escherichia coli* (*E. coli*) OP50 [65].

### 4.3. Lifespan Assay

Age-synchronized nematodes were obtained using hypochlorite treatment as previously reported [7]. All populations were raised until the L4 larvae and subsequently transferred onto new nematode growth medium (NGM) plates containing either control solvent (0.1% DMSO, as control) or the appropriate final concentrations of dissolved NOB (3.13, 6.25, and 12.5 µM). Then, 50 µM of FUDR was added to prevent viable progeny. Nematodes were transferred to new culture plates every 1–2 days and the number of live animals was monitored daily (starting from the first day of adulthood) until death. During counting analysis, animals that drilled into or escaped from the agar, or those that died of matricidal death, were excluded in time. These assays were performed in three independent trials and each experimental group included at least 109 animals.

### 4.4. Aging-Related Phenotypic Analysis

#### 4.4.1. Motility Analysis

The feeding programs were identical to lifespan assay, and on the 14th, 18th, and 22nd day of adulthood, animals were divided into three grades [17]. The criteria for judging movement were as follows: nematodes that could swim smoothly and freely were defined as A, those that started crawling only after the needle touched the head or tail were classified as C, and those between the two levels were B.

#### 4.4.2. Length and Lipofuscin Levels Alterations

At L4 larval stage, worms (*n* = 5 per group) were transferred daily to fresh treatment plates. After five days’ treatment with NOB or control solvent, animals were anesthetized with NaN_3_ (20 μM) and subsequently transferred on 1% agarose pads for determination of body length and intestinal fluorescence with a fluorescence microscope (CX23, Olympus, Tokyo, Japan). The body size and fluorescence intensity were quantified using Image J software (NIH, MD, USA). The results were expressed as a ratio of average pixel intensity in each animal’s intestine to that of control.

### 4.5. Oviposition Measurement

For oviposition measurement, L4 larvae were transferred to control or treated plates without FUDR individually. Only one worm was plated on each plate, and the parental worm was transferred every day to a new fresh separate plate without FUDR until the end of the breeding period. The resulting offspring per individual animal were kept for three days to develop into adult animals for measurement of the total progeny number, and for each concentration, five animals were conducted to calculate the average number.

### 4.6. Stress Resistance Assays

L4 larvae incubated with NOB or control plates for five days on NGM plates containing 50 μM FUDR. Similar to the above experiments, they were subsequently transferred to new plates and treated with three kinds of external stressors, respectively. These assays were repeated three times, with at least 80 nematodes per group.

#### 4.6.1. Heat Shock

On adult day 5, animals were transferred to new fresh NGM/OP50 plates and incubated at 35 °C, and monitored every 2 h thereafter until death [66]. The criteria for judging death were the same as those for the lifespan assay.

#### 4.6.2. UV Radiation

After treatment for five days, nematodes were exposed to 254 nm of UV radiation at 1200 J/m^2^ and immediately transferred to new fresh NGM/OP50 plates [47]. Thereafter, surviving and dead worms were counted daily as above.

#### 4.6.3. Oxidative Stress

Paraquat-induced oxidative stress assays were performed according to Gruber [59]. As above, on day 5 of adulthood, worms were transferred to fresh NGM/OP50 plates containing 10 mM paraquat and monitored daily as above.

### 4.7. Intracellular ROS Measurement and Detection of Antioxidant Enzyme Activity

Endogenous ROS levels in nematodes were measured with a modified DCFH-DA assay [17,67,68]. After treatment with or without NOB for five days as described above, the worms were fragmented by sonifier and the supernatant (50 μL) was added into a 96-well plate, mixing with DCFH-DA (50 μL, 100 μM). Next up, fluorescence was measured on a FilterMax F5 Multi-Mode Microplate Reader (Molecular Devices, Sunnyvale, CA, USA) at an excitation/emission wavelength of 485 nm/535 nm, while the fluorescence in distilled water was used as blank. The area under curves of fluorescence versus time was calculated to detect the ability of NOB to eliminate ROS. The rest of supernatant was collected to determine SOD activity, CAT activity, malondialdehyde (MDA) content, and protein concentration according to the manufacturer’s instruction of commercial kits (Beyotime Biotechnology, Shanghai, China).

### 4.8. Reverse Transcription-Quantitative PCR (RT-qPCR)

RT-qPCR analysis was performed to assess the gene expression. First, total RNA from about 1000 worms (treated for five days as mentioned above) was extracted using TRIzol^®^ reagent (Invitrogen, Carlsbad, CA, USA), and immediately reverse-transcribed into cDNA using the RevertAid™ First Strand cDNA Synthesis Kit (Fermentas, Hanover, MD, USA). q-PCR was performed using the Bio-Rad MiniOption™ Real Time PCR Detection System (Bio-Rad, Hercules, CA, USA) with SYBR^®^ green fluorescence dye (SYBR Green I, Cwbio Bio., Beijing, China). The relative gene expression was calculated with 2^−*ΔΔCt*^ methods using *actin-1* as the reference gene. The RT-qPCR primers used in this study are listed in Table S1.

### 4.9. Statistics

All data expressed as mean ± SD were obtained from at least three independent experiments. Survival analyses were performed using the Kaplan–Meier method by GraphPad Prism 7 software (San Diego, CA, USA) and the statistical analyses were analyzed using IBM SPSS 19 (Armonk, NY, USA). Statistical significance was determined by one-way analysis of variance (ANOVA) with Duncan’s multiple comparison post-test and differences were considered to be significant at *p* < 0.05.

## 5. Conclusions

In summary, we found that NOB could significantly exhibit beneficial effects on lifespan, healthspan, and stress resistance under normal feeding conditions of the wild type N2 *C. elegans*. Gene expression revealed that the effect of NOB on life extension and stress resistance improvement was mediated most likely through upregulation of target genes: *sod-3*, *gst-4*, *hsp-16.2*, *skn-1*, *sek-1*, and *sir-2.1*. These results indicated that NOB has potential application in the extension of lifespan and management of aging-associated diseases.

## Figures and Tables

**Table 1:** Daily foods containing nobiletin and the relative concentrations.

Species	Taxon Names	Variety	Concentration (mg/kg FW *)
mandarin oranges	*Citrus reticulata*	Nian ju	123.83–176.54
tangerines	*Citrus tangerine*	Dahongpao tangerine	1159.52–1580.75
oranges	*Citrus sinensis*	Newhall sweet orange	171.53–264.62
		Blood orange	20.56–29.02
grapefruits	*Citrus paradisi*	Changshanhuyou grapefruit	4.22–43.71

* FW means fresh weight.

**Table 2:** Effect of nobiletin (NOB) on lifespan of *C. elegans.*

Group	Number	Mean Lifespan (days) *	% of Control *	Median Survival (days)	Maximum Lifespan (days)
Control	110	24.90 ± 0.37 ^a^	100.0 ± 1.47 ^a^	25.5	31
NOB (3.13 μM)	110	27.06 ± 2.06 ^b^	108.7 ± 6.03 ^b^	29	33
NOB (6.25 μM)	109	28.61 ± 0.07 ^bc^	114.9 ± 4.34 ^b^	29	34
NOB (12.5 μM)	116	30.14 ± 0.28 ^c^	121.0 ± 1.14 ^c^	30	36

* data are expressed as mean ± SD, *n* = 3. Values with different letters in the same column indicate statistical significance (*p* < 0.05).

**Table 3:** Effect of NOB on heat shock of *C. elegans.*

Group	Number	Mean Lifespan (h) *	% of Control *	Median Survival (h)	Maximum Lifespan (h)
Control	100	13.86 ± 0.40 ^a^	100.0 ± 2.90 ^a^	14	20
NOB (3.13 μM)	90	16.40 ± 0.70 ^b^	118.2 ± 5.06 ^b^	17	22
NOB (6.25 μM)	94	16.80 ± 0.26 ^b^	121.1 ± 1.90 ^b^	18	24
NOB (12.5 μM)	96	18.10 ± 0.60 ^c^	130.6 ± 4.36 ^c^	20	24

* data are expressed as mean ± SD, *n* = 3. Values with different letters in the same column indicate statistical significance (*p* < 0.05).

**Table 4:** Effect of NOB on UV radiation of *C. elegans.*

Group	Number	Mean Lifespan (days) *	% of Control *	Median Survival (days)	Maximum Lifespan (days)
Control	113	6.88 ± 0.52 ^a^	100.0 ± 7.52 ^a^	7	11
NOB (3.13 μM)	92	7.48 ± 0.70 ^ab^	108.7 ± 10.2 ^ab^	7	13
NOB (6.25 μM)	94	8.29 ± 0.11 ^b^	120.5 ± 1.67 ^b^	8	13
NOB (12.5 μM)	97	9.17 ± 0.23 ^c^	133.3 ± 3.32 ^c^	9	14

* data are expressed as mean ± SD, *n* = 3. Values with different letters in the same column indicate statistical significance (*p* < 0.05).

**Table 5:** Effect of NOB on paraquat-induced oxidative stress of *C. elegans.*

Group	Number	Mean Lifespan (days) *	% of Control *	Median Survival (days)	Maximum Lifespan (days)
Control	85	5.06 ± 0.44 ^a^	100.0 ± 8.71 ^a^	4	10
NOB (3.13 μM)	112	5.65 ± 0.17 ^b^	111.6 ± 3.45 ^b^	6	11
NOB (6.25 μM)	81	6.60 ± 0.19 ^c^	130.5 ± 3.76 ^c^	7	13
NOB (12.5 μM)	86	7.48 ± 0.18 ^d^	147.8 ± 3.62 ^d^	7	13

* data are expressed as mean ± SD, *n* = 3. Values with different letters in the same column indicate statistical significance (*p* < 0.05).

## Supplementary Materials

The following are available online at https://www.mdpi.com/1422-0067/21/1/341/s1, Table S1: Primers used for quantitative RT-PCR analysis.
