# Supplementary material for: Nobiletin Delays Aging and Enhances Stress Resistance of Caenorhabditis elegans
Source: Int J Mol Sci. 2020 Jan 4;21(1):341. doi: 10.3390/ijms21010341 (PMC6981590; doi:10.3390/ijms21010341)
Supplement: Supplementary file 1 [file ijms-21-00341-s001.pdf]

Supplementary

Table S1. Primers used for quantitative RT-PCR analysis

Gene	Forward Primer (5′-3′)	Reverse Primer (5′-3′)
actin-1	TCGGTATGGGACAGAAGGAC	CATCCCAGTTGGTGACGATA
sod-3	GGCTAAGGATGGTGGAGAAC	ACAGGTGGCGATCTTCAAG
gst-4	ATGCTCGTGCTCTTGCTGAG	GACTGACCGAATTGTTCTCCAT
hsp-16.2	CTGCAGAATCTCTCCATCTGAGTC	AGATTCGAAGCAACTGCACC
skn-1	CACTACCGACGTCAATTTATGGAGTGTCG	ATGAAGAAGGAAGATGTTTTGTCGTGATCCG
sek-1	ATTGCCGATGGAAAGTGG	TCATTGATAAACCGAGCC
sir-2.1	ACTGAGATGCTCCATGACAATAAG	GCAAGACGAACCACACGAAC
